# A haptic-robotic platform for upper-limb reaching stroke therapy: Preliminary design and evaluation results

**DOI:** 10.1186/1743-0003-5-15

**Published:** 2008-05-22

**Authors:** Paul Lam, Debbie Hebert, Jennifer Boger, Hervé Lacheray, Don Gardner, Jacob Apkarian, Alex Mihailidis

**Affiliations:** 1 Institute of Biomaterials and Biomedical Engineering, University of Toronto, Toronto, ONT, M5S 3G9, Canada; 2 Toronto Rehabilitation Institute, Toronto, ONT, M5G 2A2, Canada; 3 Department of Occupational Science and Occupational Therapy, University of Toronto, Toronto, ONT, M5G 1V7, Canada; 4 Quanser Inc., Markham, ONT, L3R 5H6, Canada

## Abstract

**Background:**

It has been shown that intense training can significantly improve post-stroke upper-limb functionality. However, opportunities for stroke survivors to practice rehabilitation exercises can be limited because of the finite availability of therapists and equipment. This paper presents a haptic-enabled exercise platform intended to assist therapists and moderate-level stroke survivors perform upper-limb reaching motion therapy. This work extends on existing knowledge by presenting: 1) an anthropometrically-inspired design that maximizes elbow and shoulder range of motions during exercise; 2) an unobtrusive upper body postural sensing system; and 3) a vibratory elbow stimulation device to encourage muscle movement.

**Methods:**

A multi-disciplinary team of professionals were involved in identifying the rehabilitation needs of stroke survivors incorporating these into a prototype device. The prototype system consisted of an exercise device, postural sensors, and a elbow stimulation to encourage the reaching movement. Eight experienced physical and occupational therapists participated in a pilot study exploring the usability of the prototype. Each therapist attended two sessions of one hour each to test and evaluate the proposed system. Feedback about the device was obtained through an administered questionnaire and combined with quantitative data.

**Results:**

Seven of the nine questions regarding the haptic exercise device scored higher than 3.0 (somewhat good) out of 4.0 (good). The postural sensors detected 93 of 96 (97%) therapist-simulated abnormal postures and correctly ignored 90 of 96 (94%) of normal postures. The elbow stimulation device had a score lower than 2.5 (neutral) for all aspects that were surveyed, however the therapists felt the rehabilitation system was sufficient for use without the elbow stimulation device.

**Conclusion:**

All eight therapists felt the exercise platform could be a good tool to use in upper-limb rehabilitation as the prototype was considered to be generally well designed and capable of delivering reaching task therapy. The next stage of this project is to proceed to clinical trials with stroke patients.

## Background

The quality and ability of a person's reaching motion is important as it fundamental for many activities a person needs to be able to perform if s/he is to be independent, such as dressing, eating, and getting into/out of a chair. Additionally, the ability to reach enables support and anchoring to increase an individual's safety and mobility [1]. Having a stroke can reduce a person's ability to reach because of the resulting death of associated brain cells. Fortunately, due to the plasticity of the brain, at least partial recovery is usually possible [2]. Furthermore, recovery can be greatly enhanced by rehabilitation therapy [3].

### Rehabilitation therapy

Rehabilitation therapy after a stroke is crucial to helping the survivor regain as much use of his/her limbs as possible. In particular, intervention intensity and specificity have been shown to have a profound effects on the recovery of the stroke patient.

#### Intervention intensity

Studies with constraint-induced therapy, whereby the patient's unaffected upper-extremity is constrained for long periods of time to force the person to use their affected upper extremity, suggest that there are benefits to drastically increasing the patient's training intensity, in particular increasing the number of hours of consecutive therapy seems to have a large, positive impact on recovery [3,4]. These studies also report that increased usage of the affected limb provide long term benefits even when implemented after recovery has plateaued in the chronic phase (i.e. more than 1 year from occurrence of stroke) [3,5]. Moreover, short term studies using constraint-induced therapy on sub-acute stroke patients also show promising results [6,7]. Thus, it would seem that it is in the best interest of the patient to engage in rehabilitation training that is as intense and frequent as is safely possible.

#### Intervention specificity

Alexander *et al*. investigated the effects of task-specific resistance training with physically impaired older adults. The study trained 161 subjects in bed and chair-rise tasks over a 12 week period. Their findings concluded that task-specific resistance training increased the overall ability and efficiency of the subjects [8]. So although it is well established that practice is needed for motor learning to occur (e.g. [9]), giving a patient a specific task to perform may encourage greater compliance and success in a rehabilitation intervention. A literature review by Page cites studies of task-specific training protocols at various intensities that have induced lasting cortical and functional changes in stroke patients [10].

### The use of haptic-robotics in therapy

A haptic interface is a human-computer interface that uses the sense of touch. The sense of touch is unique in that it can allow for simultaneous exploration and manipulation of a particular interface [11]. By applying forces on the operator, a haptic device gives the tactile sensation of interacting dynamically with physical objects. Motor skills recovery is dependent on both afferent and efferent stimulation [12], thus the capability of a haptic feedback system for simultaneous exploration and manipulation makes it ideal to use with stroke rehabilitation therapy. Consequently, there has been a recent rise in popularity of haptic feedback in therapy, and the devices that have been used are yielding encouraging results. Lum *et al*. designed a novel therapy and assessment device that passively and actively guided users through upper-limb movements and recorded their performance [13]. Krebs, Volpe, *et al*. have contributed a large amount of data from clinical trials with MIT-MANUS and other robots that show improvements in patient outcomes when upper-limb training is present [14,15]. Loureiro *et al*. strove to achieve a low cost modular home based system through GENTLE/s, a haptic and virtual reality system for upper-limb stroke rehabilitation [16]. Reinkensmeyer *et al*. used a different approach by exploring the simplicity of reaching motion therapy constrained to a straight line through the implementation of their Assisted Rehabilitation and Measurement Guide (ARM Guide) [17]. Rosati *et al*. devised MariBot, a 5 degree of freedom (DoF) system for bed-side therapy with acute period stroke patients [18]. Nef and Riener developed ARMin, a large semi-exoskeleton with 6 DoF [19]. For further details and a comparison of robitc-aided upper-limb rehabilitation, the reader is referred to [20].

Compared to the robots mentioned above, the ARM Guide is the one that is most similar to this project [17]. First of all, most of the systems above are quite large (many of them hospital based), operate as an exoskeleton to the user's arm, and/or require constant therapist supervision to ensure absolute user safety. Secondly, the reaching motion supported motion of the ARM guide is quite similar to the device created in this researh. The ARM Guide constrains the user to one simple 3 DoF reaching motion (a passive, linear reaching motion with adjustable yaw and pitch using locking mechanisms), however, this is coupled with sensors such as the 6 DoF force/torque sensor on the splint bearing to monitor abnormal tone, spasticity, and lack of coordination. In fact, Reinkensmeyer *et al*. stated that one of the first objectives of the ARM Guide is to provide an improved diagnostic tool for assessing arm movement tone, spasticity, and coordination after brain injury [17]. Although assessment and client performance are important factors, the primary focus of the research below is to construct a tool for (possibly long-term) post-stroke, upper-limb rehabilitation training.

The new robotic system described in this paper will provide several advantages over the current state-of-the-art. Firstly, the system will be lighter and more compact, allowing it to be used in various contexts and locations, such as at the patient's bedside, anywhere in a clinic, or at home. It will also be more intuitive and simpler to use as it does not require the user to have to learn how to "interact" with complex hardware. Finally, it will be capable of autonomous guidance through the use of a artificial intelligence based controller, which will allow the system to make decisions with respect the type of exercise automatically based on real-time feedback from the system and operator. This last advantage and the algorithms that have been developed will be the basis of a future publication. It is expected that the combination of the advantages above will result in a system that is versatile and accessible in a variety of settings.

Patients usually start with about 60 to 70 degrees of flexion in the elbow. The movement takes place in the saggital plane with the hand in alignment with the shoulder. The hand is pushed forward until it reaches the final desired position and then follows the reverse path until the hand and arm return to their initial positions. It is important to note that the motions should be smooth and controlled while the person performing the exercise maintains an upright posture. There are variations to this movement that are progressively implemented as the patient begins to regain use of his/her limb. One variation of this forward movement is to direct the path laterally outward at approximately 45 degrees using shoulder abduction and rotation on the horizontal plane. In the event that the patient requires assistance extending the elbow while exercising, gentle cueing is provided by the therapist using his/her fingertips to gently touch the patient between the ulna and radius (two long forearm bones) just below the olecranon (elbow), as well as portions of the triceps brachii tendon just above the olecranon. The therapist moves his/her touch away from the elbow to provide as much stimulation as possible. This touch is for directional cue and stimulation, not actual movement assistance, and therefore should be barely pushing the limb.

## Purpose and objectives

The motivation for this research and to develop a new rehabilitation robotic system stemmed from preliminary discussions with several occupational and physical therapists who identified various challenges in providing rehabilitation to their clients. From these preliminary discussions, a primary concern that was identified was the inability to provide "around the clock" access to exercise therapy for their stroke patients in order to increase training frequency, and subsequently, positive rehabilitation outcomes. Therapists also identified the opportunity for a technological solution that can help support a labor-intensive task, thereby enabling them to focus on other aspects of a patient's recovery or treat multiple patients at a time. This would in turn help to reduce patients' dependence on therapists with respect to their rehabilitation plans and exercise, which becomes particularly important when patients leave the clinic and need to continue with their rehabilitation at home. This ability to perform the necessary exercises at home was identified by the therapists as one of the greatest potentials for a new robotics-based system.

The authors also discussed with the therapists the task they felt was crucial to successful patient rehabilitation but has little or no equipment-based support. The therapists identified the action of reaching forward as one of the most fundamental to independent self-care and safety. The basic reaching motion begins with a slight forward flexion of the shoulder, extension of the elbow, and extension of the wrist with contact on a surface by the hand. Patients usually start with about 60 to 70 degrees of flexion in the elbow. The movement takes place in the saggital plane with the hand in alignment with the shoulder. The hand is pushed forward until it reaches the final desired position and then follows the reverse path until the hand and arm return to their initial positions. It is important to note that the motions should be smooth and controlled while the person performing the exercise maintains an upright posture. There are variations to this movement that are progressively implemented as the patient begins to regain use of his/her limb. One variation of this forward movement is to direct the path laterally outward at approximately 45 degrees using shoulder abduction and rotation on the horizontal plane. In the event that the patient requires assistance extending the elbow while exercising, gentle cueing is provided by the therapist using his/her fingertips to gently touch the patient between the ulna and radius (two long forearm bones) just below the olecranon (elbow), as well as portions of the triceps brachii tendon just above the olecranon. The therapist moves his/her touch away from the elbow to provide as much stimulation as possible. This touch is for directional cue and stimulation, not actual movement assistance, and therefore should be barely pushing the limb.

Using this motivation, the purpose of this research was to develop and evaluate an easy-to-use, intuitive haptic robotic device that could deliver upper-limb reaching therapy to moderate-level (Chedoke-McMaster stage 4 [21]) stroke patients. The long term goal of this project is to develop a device that employs artificial intelligence to autonomously customize the exercise (e.g. applied force and number of repetitions) to the client and delivers it through an engaging haptic interface that provides the client with safe, effective, motivating, and challenging rehabilitation. The artificially intelligent interface would also allow the clinician and client to access data regarding progress/setbacks and react to these accordingly. However, before the design and implementation of highly specialized artificial intelligence algorithms can begin, the intended hardware must be selected. It is crucial to test a prototype device with trained experts in order to evaluate device operation, safety, and efficacy. The following sections present the design of a prototype rehabilitation device and its evaluation by physical and occupational therapists who had experience in post-stroke, upper-limb rehabilitation.

The objectives of this research were to evaluate:

1. What are the design requirements for a self-contained haptic-robotic device for moderate level (Chedoke-McMaster stage 4 [21]) upper-limb reaching task stroke rehabilitation?

2. Can an active, two DoF haptic-robotic device emulate a weight bearing reaching motion therapy?

3. Can unobtrusive sensors detect abnormal postures during reaching motion?

4. Can this robotic device deliver reaching task therapy without restraining the user?

5. Can basic actuators provide provisional stimulation/cuing forces for reaching task therapy?

## The upper-limb rehabilitation prototype

**Figure 1:**
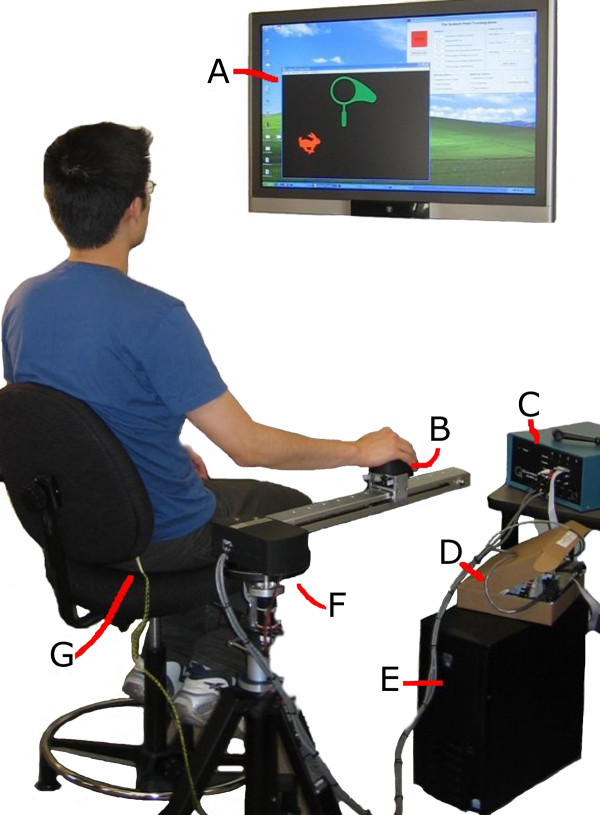
**Upper-limb post-stroke rehabilitation system in use**. The system consists of a (A) visual display, (B) end-effector with wrist sensor, (C) power amplifier, (D) terminal board, (E) computer, (F) haptic-robotic system, and (G) trunk sensors on chair back.

After the forward reaching motion was identified as the target exercise, the researchers worked with three professional therapists to create the prototype design. There are four main components to the prototype system: 1) The haptic-robotic device, which emulates the weight bearing motion using haptic feedback; 2) the postural sensor, which identifies upper body posture abnormalities during the exercise; 3) the elbow stimulation device, which provides provisional stimulation to the elbow when needed; and 4) the computer interface, which gives visual feedback to the user. Figure 1 shows a picture of the final prototype system in use.

### Haptic-robotic exercise platform

End-effector based rehabilitation robots are commonly located in front or to the side of the user such that the robotic arm points toward the person. This positioning is to ensure safety and maximize range of motion, as the robot and the operator occupy mutually exclusive spaces. Controlling a robot in this position requires an added layer of difficulty in calculating the kinematics and dynamics involved. But if the axis of motion of the robot and user are aligned, then controlling the robot can be greatly simplified as variables that describe the robot's motion correlate to user movements. For example, one DoF could correspond with the shoulder traversal movement and another DoF can correspond to shoulder flexion/extension. Moreover, appropriately powered motors can be used for each axis because the DoFs are decoupled. This can greatly reduce the size and cost of the robot.

**Figure 2:**
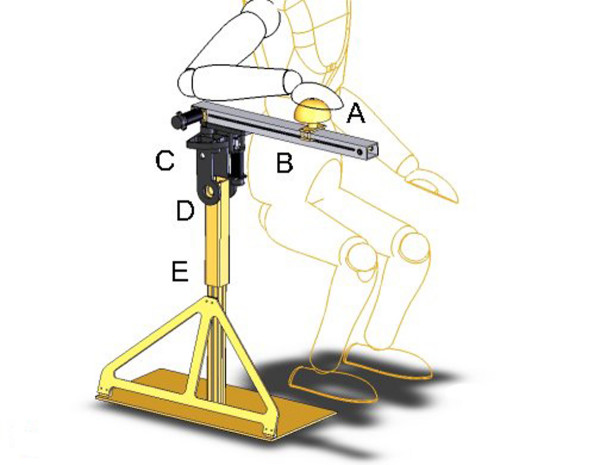
**Schematic of the final design concept**. Features include the (A) end-effector, (B) linear track, (C) traverse motion, (D) pitch adjustment, and (E) height adjustment.

After several iterations of our design process, which produced various concept ideas [22], our final system design (see Figure 2) was produced in collaboration with our industrial partner, Quanser Inc (Markham, Canada). In this prototype, a motor drove a belt and two gear system to translate rotational motion to linear motion of the end-effector. Another motor located at the elbow of the device actuated the swing of the robotic arm, which had a lateral range of -20 to 160 degrees from the saggital plane. This range ensured the device would not to hit the person using the device while still providing a wide range of shoulder horizontal abduction. Some features of the haptic device are:

• 2-dimensional actuated range of motion

• Non-restraining (i.e. the user is not attached to the device in any way) for better usability, freedom of movement, and safety

• Range of motions for various exercises other than reaching

• Adjustable for different statures

• Simple functionalities

• Replaceable end-effector

• Less than 10 kg total weight

• Collapsible design for storage and transportation

The haptic controller was developed by the project's industry partner, Quanser Inc. The controller was an impedance based design whereby the position of the end-effector determines the force feedback by the robot, as described in more detail by Hogan [23]. To increase safety, a light-sensitive diode was added to turn off power to the end-effector as soon as the user removed their hand. The end-effector's speed was also limited by software for extra safety. It should be noted that for this particular prototype the haptic controller only provided three magnitudes of damping (or resistance) on the end effector and linear track (as shown in Figure 2)-10 Ns/m, 50 Ns/m, and 100 Ns/m, which were manually selected via the user interface. The eventual final haptic controller will include an artificial intelligence based controller that will automatically adjust these resistance levels in real-time as user performance changes, as would happen with a human therapist. A description of the full haptic controller will be the topic of a future publication.

### Posture sensing system

**Figure 3:**
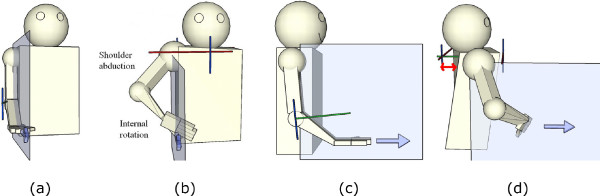
**Common compensatory strategies during the reaching exercise**. Stroke survivors often exhibit abnormal shoulder abduction/internal rotation and trunk rotation during the reaching task. (a) Front view of normal reaching, (b) front view of abnormal reaching, (c) side view of normal reaching, and (d) side view of abnormal reaching.

Stroke survivors commonly compensate for limited upper-limb movement with upper body (trunk and upper extremity) motion. Compensatory motions include shoulder abduction and internal rotation, and flexion/rotation of the trunk when reaching, as illustrated in Figure 3[24]. The presence and severity of these reaching abnormalities are an important indicator of the quality of the movement and the patient's overall ability level [21,25]. During rehabilitation, it is important to discourage these movements so that the patient learns to reach properly with their arm, resulting in better overall functionality.

#### Trunk flexion/rotation detection using photo-resistors

If the patient is seated in a chair with their back resting on the chair-back normally, bend resistors or photo-sensitive resistors can be used to detect the resulting gap between the chair and the patient when trunk rotation occurs. Photo-sensitive resistors were chosen for the final prototype design because they are less intrusive, smaller in size (5 mm in diameter and 2.4 mm thick), inexpensive, and easy to setup and use.

**Figure 4:**
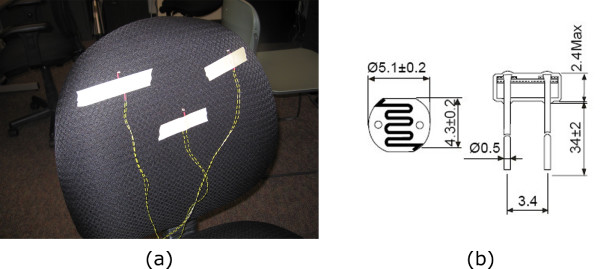
**Sensors used to detect abnormal trunk movement**. Photo-sensitive resistors were used to detect when a the user had abnormal trunk movement. (a) Placement of sensors on the chair back and (b) specifications of the photo-resistor.

As shown in Figure 4a, a total of three sensors were used, with one photo-resistor placed behind each of the lower left and right scapulas, and the lower back. This was to distinguish between left and right rotation and more severe flexion (which displaces the lower back). The sensitivity of the photo-sensitive resistors were set to detect a gap of approximately 2 cm. This meant that if the person's back was 2 cm or more from the photo-sensitive resistor (and therefore chair) they were considered to be sitting with an abnormal posture. This high sensitivity was used at this stage because correct posture during the reaching task is important for a successful rehabilitation outcome and ideally the client should not use move their trunk forwards to complete the reaching task. However, many potential users of the device will not be able to achieve this goal, therefore in a clinical situation the therapist should determine what threshold is appropriate for each individual.

#### Shoulder abduction and internal rotation detection using end-effector rotation

**Figure 5:**
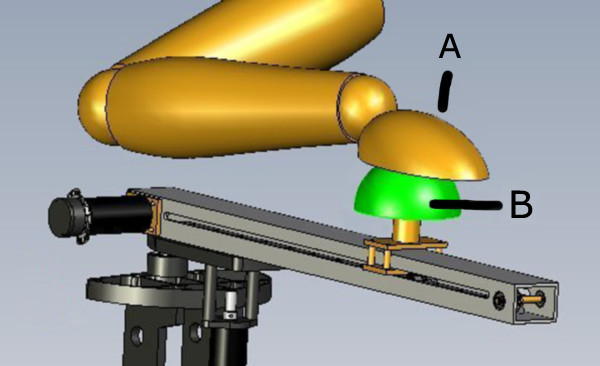
**Design of the end-effector used in prototype trials**. The (A) end-effector was designed to rotate freely, placing the challenge on the user to practice controlling their upper-limb. The amount of rotation of the end-effector can be translated into amount of shoulder abduction and internal rotation.

The biomechanics of the upper-limb cause a rotation in the wrist and hand when there is shoulder abduction and internal rotation [26]. The end-effector of the prototype was designed to rotate independently of the motion of the robot and the direction of the exercise, as shown in Figure 5. A rotation of the end-effector corresponds to undesired shoulder abduction/rotation. The rotation of the end-effector was monitored in real-time and if the rotation was greater than a preset threshold, empirically set to 15° in the prototype, then the movement was designated as abnormal. This 15° threshold was determined by having a therapist use the system, rotating the end effector in increments of 5° until the therapist decided the posture was abnormal. This process was repeated until it was clear which degree increment best signified the threshold between a normal and abnormal posture with respect to shoulder abduction and internal rotation. This 15° tthreshold was then set in the system's software and compared in real-time with the value generated by the end effector. For this prototype the same threshold value was used for all users/subjects, although the authors are aware that eventually this value must be able to be altered by the overseeing therapist to reflect each user's abilities. When the postural sensors detect an abnormality, a visual and auditory prompt is provided on the graphical user interface reminding the user to correct his/her posture. These prompts continue until the user has rectified his/her posture. Presently the detection of an abnormal posture is simply to provide statistics for the therapist and reminders for the user and therefore does not affect the decisions made by the system (e.g. changes in the targeted reaching distance or strength), although this is being considered for a future version of the system.

### Elbow stimulation using vibration

At the request of the therapists, the subject's hand was not restrained to the device in any way. This means that the the system could only provide resistive exercise, namely the haptic device was intentionally designed so it could not physically pull the person to reach farther. A stimulation device was added to stimulate the elbow extensor muscles to emulate the current practice where the therapist provides provisional stimulation by a gentle outward stroking of the patient's triceps brachii tendon and anconeous muscle, as described in section *The Reaching Exercise*. This stimulus would only be provided if the system detects that the user is having difficulty reaching the designated target and is intended to provide a gentle tactile prompt to encourage the user to try and reach a bit further. As the elbow stimulation is intended as a physical form of "encouragement", it would only be activated by the system if necessary, with the initiation and duration of the stimulation based on the interface/game that the user is interacting with. For example, as described later in this paper, one of the interfaces is a game where the user must move a cursor to a target. In this case, if the person cannot quite reach the target or has trouble initiating the reaching movement towards it, then the elbow stimulus would be activated and turned off once the user begins the movement.

**Figure 6:**
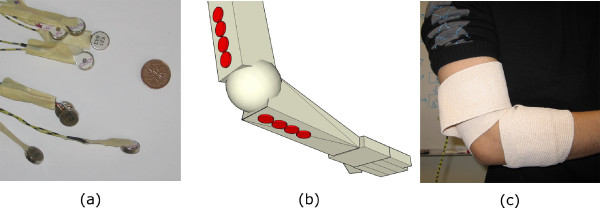
**Elbow stimulator**. (a) Eight vibration cells were positioned along (b) the posterior side of the elbow with four cells lined above and four cells lined below the elbow. For the prototype, the cells were (c) attached to the user with a lightly-bound compression bandage.

A previous study with cutaneous vibratory stimulation on eight spinal cord injured subjects showed isometric strengthening of elbow extension [27]. Therefore, rather than striving to imitate the therapists' actions exactly, our intention was to use the therapists' actions as a guideline with respect to the type of stimulation that may be effective. As such we experimented with using vibration to stimulate the patient's elbow, as this is a simple, versatile, cost-effective, and previously proven approach (albeit with a different user group). It was hypothesized that using a series of vibration cells activated synchronously would provide sufficient sensory stimulation to bring the stroke patient's attention to appropriate muscles that they needed to contract. Eight vibration cells (manufactured by JinLong Machinery, model #C1234B016F [28]) were placed along the posterior side of the arm, with four cells above and four cells below the elbow, as demonstrated in Figure 6b. Each sequence of activation would provide stimulation by firing the pair of cells closest to the elbow, followed by firing the next closest pair and turning off of the first pair, and so on. For the prototype, the vibration motors were attached to the subject using a tensor bandage as in Figure 6c. As this was a preliminary attempt to gain some insight from professionals regarding the perceived usefulness of vibrational elbow simulation, precise positioning was not necessary.

### Human computer interface

**Figure 7:**
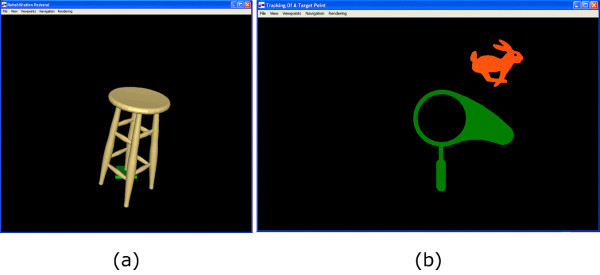
**Interfaces for rehabilitation prototype**. Screen-shots of the display for the (a) virtual stool and (b) rabbit-catching game interfaces.

The virtual environment for this prototype was developed by our industry partner, Quanser Inc. The computer interface for the prototype used a monitor to display a representation of what the haptic system was rendering. Stools are commonly used by therapists as a tool to keep a patient's hand steady during reaching motion therapy therefore the first interface showed a virtual stool, as shown in Figure 7a. The intention of this exercise was to have the user manipulate the haptic end-effector while feeling dynamic physical forces based on the virtual stool's orientation. For example, when the stool looked like it was tilted at a large angle, the person could feel an outward force in the corresponding direction. This force was proportional to the angle of the stool, with larger angles (i.e. "falling over" further) producing larger forces. The second interface, shown in Figure 7b, has a simple cursor (a net) and a target (a rabbit). The location of the end-effector in the plane of motion was represented by a corresponding movement of the net on the screen. The goal of this task was to move the net using the end-effector and "catch" the rabbit. To encourage dfferent types of reaching motions, the location of the rabbit can be randomized or pre-determined using cartesian coordinates.

For both interfaces, several settings could be changed, such as damping of the movement and attractive or repulsive forces near the target. Virtual boundaries could be set so the user would feel as if they were pushing against a stiff wall on off-axis movements, intended to enable some users to concentrate on training just a basic reaching movement with restricted side-motion freedom.

## Methods

### Participants

Pilot trials with the new robotic system were conducted with clinician-users, as opposed to with client-users (i.e. actual stroke patients) because of safety and ethical concerns. As the device was a new, untested technology, trials with a healthy, expert population were necessary to assess the device, ensure that all the system's features worked as intended and that all potential risks were eliminated. As such, these trials used professional occupational and physical therapy clinicians to test the robot's features and capabilities, relying on their expertise to assess if the system is appropriate for use by stroke patients in a subsequent study.

To be eligible for participation in this study, the clinician-participant had to:

1. be a practicing physical or occupational therapist,

2. have at least one year of experience with upper-limb stroke rehabilitation,

3. not be involved with the development of the system, and

4. read the information sheet and sign the consent form (both documents were approved by the Toronto Academic Health Sciences Council and the University of Toronto Health Sciences Research Board).

Eight therapists (all female) from local rehabilitation hospitals participated in this study. Four were physical therapists and four were occupational therapists and had an average of 4.0 years (SD 2.6, range = 1 to 8 years) of experience with upper-limb stroke rehabilitation. All participants held university level degrees (either at an undergraduate or graduate level). None of the participants were actively involved in research.

### Procedure

System usability was gathered through a semi-structured interview format, which included a combination of 4-point Likert scale and open-ended questions. Questions were worded to elicit responses as a measurement of the participant's satisfaction and were rated on a Likert scale of one to four (with a one representing bad, two representing slightly bad, three representing slightly good, and a four representing good). Appropriately corresponding adjectives were used in place of good or bad for each question, for example, "With low power, how comfortable (4) or uncomfortable (1) is the system to use?". A semi-structured interview was used in order to elicit responses to the open-ended questions and to allow the participants to answer questions while they actually used the robotic system. Furthermore, while there are limitations associated with using a 4-point Likert scale (as opposed to a 5 or more point scale), the authors wanted to use a simpler scale since the clinicians would be providing assessments while using the robotic system at the same time, therefore a simple scale allowed for ease in evaluation in the semi-structured interview approach. Furthermore, since a primary objective of these responses was to identify design changes required to improve the safety of the system, it was important that to ensure that the evaluation elicited an opinion (whether positive or negative) from each participant. Thus, a "neutral" response, which can often be found in higher-point scales, was not included.

A limitation of this procedure was the need to employ a previously untested usability questionnaire. To address this limitation, the questionnaire was developed and piloted with a human factors and usability expert to ensure to the questionnaire capture the desired data. The questionnaire was then piloted with two clinician-subjects who were not involved in its design or in the study itself. Subsequent refinements to the questionnaire were made with assistance from the human factors and usability expert.

### Analysis of data

Data were analyzed using descriptive statistics.

## Results

Table 1 summarizes the participants' responses regarding several features of the haptic exercise device. Each participant performed the same reaching motion at several different damping, or resistance (difficulty), levels of the exercise. The therapists were asked to rate various characteristics of the haptic device in terms of their own perceptions as well as their professional opinion with respect to stroke patients. Table 2 shows the mean and standard deviations of the therapists' responses to comfort, perceived safety, and quality of motion of the device at no (0 Ns/m), low (10 Ns/m), medium (50 Ns/m), and high (100 Ns/m) damping settings. In particular, the participants were asked to rate their opinion on "Do you think this maximum resistance is too weak (1) or strong enough (4) for use in therapy?". On a Likert scale from one (too weak) to four (strong enough), the therapists rated the device's strength as a mean of 3.8 and standard deviation of 0.5.

**Table 1:** Therapists' ratings of various prototype features.

	Range	Resemb.	Setup	Removal	Handle	Power	Comfort	Safety	QOM
Mean	3.3	3.2	2.8	3.0	2.4	3.8	3.4	3.7	3.6
SD	0.5	0.4	0.7	0.8	0.5	0.5	0.7	0.6	0.6

Mean and standard deviation (SD) of therapists' responses to the use of the haptic platform in regards to the *range *of motion, movement *resemblance *to traditional therapy, *setup *procedure difficulty, *removal *procedure difficulty, prototype's *handle *(i.e., end-effector), resistance *power *sufficiency, *comfort *level, perceived *safety*, and quality of motion (*QOM*). Ratings are from 1 (bad) to 4 (good), N = 8.

**Table 2:** Therapists' ratings of prototype operation.

	No Power		Low Power		Medium Power		High Power	
	Mean	SD	Mean	SD	Mean	SD	Mean	SD
Comfort	3.75	0.46	3.75	0.46	3.63	0.52	3.63	0.52
Comfort-P	2.69	1.28	3.13	0.99	3.50	0.76	3.38	0.74
Safety	4.00	0.00	4.00	0.00	3.88	0.35	3.75	0.71
Safety-P	3.25	1.16	3.63	0.74	3.63	0.74	3.38	0.92
QOM	4.00	0.00	3.75	0.46	3.38	0.74	3.38	0.92
QOM-P	3.75	0.71	3.88	0.35	3.19	0.84	3.06	0.78

Mean and standard deviation (SD) of haptic platform responses for *comfort *level, perceived *safety*, and quality of motion (*QOM*) with respect to therapists' own experience with the prototype and their opinion of prototype suitability for use with stroke patients (denoted by -P suffix). Ratings are from 1 (bad) to 4 (good), N = 8.

To test the posture system, the participants were asked to perform a normal forward reaching movement, a normal reaching outward movement, and two different abnormal forward movements of the trunk. Each movement was repeated three times (for a total of 12 movements by each participant). The results for the trunk sensors are presented in Table 3. Similar to the trunk tests, the participants were asked to perform a normal forward reach, a normal lateral outward reach, and two abnormal forward reaches with shoulder abduction and/or internal rotation. Each movement was repeated three times (for a total of 12 movements by each participant). Conditions where the end-effector rotated above the predetermined threshold of 15° or more (i.e. abnormal) were recorded by the system. Results from the tests are presented in Table 4. Table 5 presents the mean and standard deviation of the participants' responses regarding the elbow stimulation device. The participants were asked for their preferences and dislikes of the computer interface.

**Table 3:** Trunk sensor performance.

Sensor Response	Body Movement	
	Abnormal	Normal
Detection	48	3
No detection	0	45

Performance of the trunk sensors during therapist simulation of normal (N = 48) and abnormal (N = 48) reaching motions.

**Table 4:** Detection of movement type through end-effector rotation.

Sensor Response	Body Movement	
	Abnormal	Normal
Detection	45	3
No detection	3	45

Detection of end-effector rotation above the preset threshold of 15° for therapists simulating normal (N = 48) and abnormal (N = 48) movements.

**Table 5:** Therapists' ratings of the elbow stimulation device.

	Importance	Effectiveness	Stimulation	Alternative
Mean	3.9	2.3	2.4	2.5
SD	0.4	1.2	1.1	1.3

Mean and standard deviation (SD)of therapists' ratings of the elbow device regarding the *importance *of stimulation during traditional therapy, *effectiveness *of the elbow stimulation device, *stimulation *of reaching motion by the device, and option of using the device as an *alternative *when a therapist is not present. Ratings are from 1 (bad) to 4 (good), N = 8.

All eight of the therapists preferred the target tracking (rabbit) game to the stool simulation because it was "intuitive", "engaging", and "motivating", whereas the stool stimulation was "boring" and "lacks purpose".

## Discussion

### Haptic exercise platform

Participant feedback regarding the haptic exercise platform was encouraging, with seven of the nine categories having a mean score of more than a 3.0 out of 4.0. In addition, comments from the therapists were very positive. Two or more therapists commented favorably on the following aspects:

1. various operating positions

2. wide range of shoulder motion

3. focus on the lateral exterior range

4. switchable end-effector

5. ease of use

Through therapist feedback, it also became evident that the two aspects that need more attention are the supporting structure and the end-effector. As seen in Figure 1, the design of the prototype base caused the device arm, and therefore the end-effector, to be higher up than originally anticipated. This resulted in the operator sitting in a chair with the seat further from the ground than conventional chairs. Also, the tripod base causes the position of the device to be farther away from the body than desired and may prohibit the use of a wheelchair. These deficiencies are reflected in the relatively lower "ease of setup" score. To correct these issues, a new base should be designed that lowers the device and has a less sideway obtrusion without compromising safety or stability.

Participants commented that the end-effector used in the prototype trials could be used in some, but not all stroke rehabilitation cases. In particular, the therapists indicated that many stroke patients would find it difficult to maintain their hand on the end-effector during the exercise, therefore the lack of a mechanism to secure the hand of the user on the end-effector is likely to hinder with the device's usability. Having a variety of switchable end-effectors is an important feature because therapists would like more options available to address to the different needs of the patients.

While the therapists rated some aspects of these different damping/power settings, the primary goal of this stage of research was to ensure that the robotic system and its features are appropriate for upper-limb stroke rehabilitation and can be used safely and effectively at each setting. More in-depth testing of the final haptic controller and interface will be completed in a future study

### Postural sensing system

It was found that when a user was leaning to one side the sensor on the other side of the scapula was exposed. When a user was slouching, there was excessive curvature of the spine and either the upper sensors or the lower sensor were exposed. Because of this, the posture sensor was able to detect therapists leaning forward, leaning to the side, and slouching and the actual posture of the user could be determined by identifying exposed sensor(s).

As seen in Table 3, the true positive rate (sensor firing during abnormal posture) and true negative (no sensor firing during normal posture) rate of the trunk senors for trunk flexion and/or rotation were 100% (48 of 48) and 93% (45 of 48) respectively. While there was good detection of these postures, the therapists stated that they would also like to know the severity of the abnormal trunk postures rather than just a binary output. Possible ways to achieve this include: 1) replacing the photo-resistors with distance sensors to measure the displacement of the trunk; 2) using a pressure sensing pad on the chair seat to estimate the center of gravity of the user; or 3) using a camera and artificial intelligence to monitor the trunk or the entire upper body.

As shown in Table 4, the true positive rate and true negative rate of the end-effector sensor were 94% (45 of 48) and 94% (45 of 48) respectively. Although these results are a good start, the limitation with this approach is that the system can only detect shoulder abduction and internal rotation; the upper-limb has many degrees of freedom and many more abnormalities are possible. To improve on the upper-limb sensor, there are a few modifications that could be made to the current prototype. One possibility is to modify the detection algorithm by adding longitudinal movement of the end-effector to the presently measured rotation value, allowing a more accuarte estimate of the upper-limb posture. For even greater accuracy and flexability, an kinematic vision system could also be used to monitor the upper body.

### Elbow stimulation device

Generally, the feedback regarding the vibrational elbow stimulation device were unfavorable. However, therapist feedback did yield new ideas and specifications to consider, such as flexible positioning of the vibration cells such that therapists themselves can decide on the appropriate positioning for each patient. However, whether this component is worth further investigation is debatable at the moment. A few of the therapists said they were satisfied with the system for use in therapy without the elbow stimulation mechanism. Therefore, the application of this component is a low level priority as it seems to add little value in its present form.

### Computer interface

The therapists were more interested in testing the software interface than any other part of the system. This could be because the interface is what provides the method of interaction or because participants were not instructed how to perform tasks and were just asked to explore the games freely. The therapists were enthusiastic about the possibility of software interfaces that provide visual feedback and motivation to the user. As the reaching task is very repetitive, patients can lose interest in the exercise quickly. The therapists felt that having more games and options would allow for more task flexibility and incorporation of personal preferences. It is important to remember that even though these interfaces can be labeled as 'games', they truly are part of a rehabilitation system. Therefore, in addition to the entertainment value, therapists felt that these interfaces could be more useful by covertly incorporating more precise therapy techniques. For example, an isometric exercise that has the user pause at certain positions during a reaching motion can be good for recovery. Encouragingly, the therapists' focus on the content of the "games" seems to be an indication that the device was effectively interacting haptically with the images on the monitor, hence the focus was on the computer task rather than the operation of the device.

## Conclusion

This paper presents a preliminary design and evaluation study to examine the feasibility of a portable, affordable, non-invasive haptic robotic upper-limb rehabilitation device. From the study, the researchers have come to the following conclusions:

1. Supported by data from preliminary testing with experienced professional stroke rehabilitation therapists, the researchers feel a reasonable design specification has been developed. With the recommended modifications, the system should be suitable for testing with stroke patients.

2. From the results and discussions, it was demonstrated that the haptic exercise platform was capable of performing reaching task exercises.

3. Unobtrusive postural sensors were shown to be able to detect trunk flexion and rotation, shoulder abduction and internal rotation during reaching exercise in lab conditions. The limitation for this postural sensing system is that it only provides a binary output to its targeted abnormal postures. Another limitation is that the targeted abnormal movements represent just a portion of all the typical abnormalities manifested by stroke patients during therapy.

4. Although the haptic-robotic device is able to deliver reaching task therapy without restraining the subject, it is essential that the therapist is given the option to secure the user's hand to the end-effector.

5. The vibration elbow stimulation device was not found to be useful. This is thought to be because of the method of implementation and the lack of versatility for each individual's needs. However, it was also found that the rest of the upper-limb rehabilitation system is acceptable for use in therapy without the inclusion of the stimulation device.

Although previously designed systems, such as [17], focus on reaching motion therapy, the robotic platform described in this paper may be more advantageous because it is relatively lightweight (as compared to other existing systems), it has the potential to be scalable to other exercises for the upper body, and it may be more intuitive to use has it has less features and components than other systems. Additionally, the system presented here includes a postural sensing component to observe upper body compensation during the reaching task, which is a common phenomenon in stroke patients. Although these results are promising, in-depth trials with actual stroke patients will be needed before these conclusions can be stated more definitively.

With the modifications identified through this study and the addition of the new artificial intelligence based haptic controller, it is hoped that this system will eventually: 1) empower patients to choose when and where they want their exercise therapy; 2) support therapists in a labour intensive task; 3) reduce dependency on hospital resources; and 4) assist in re-integrating stroke patients back into the community.

## Future work

The researchers have began to make the recommended modifications to the system. Once modifications are complete, a new artificial intelligence controller will be added to make real-time decisions during the exercise session based on the real-time feedback from the system [29]. It is hoped the addition of this controller will further reduce the need for therapist intervention during therapy and will provide consistent, accurate control during rehabilitation. Therapist-supervised clinical trials with stroke patients using the rehabilitation device are expected to commence in 2008.

## Competing interests

The authors declare that they have no competing interests.

## Authors' contributions

PL and Quanser Inc. developed the proposed system and study design, performed the trials, completed the data analysis, and drafted the manuscript. AM supervised the project. All authors participated in the conception and design of the system, and in the preparation of the final manuscript.
